# Efficacy of Intercostal Nerve Block for Pain Control After Percutaneous Nephrolithotomy: A Systematic Review and Meta-Analysis

**DOI:** 10.3389/fsurg.2021.623605

**Published:** 2021-01-28

**Authors:** Tao Chen, ZhenQiang Zhu, Jianlong Du

**Affiliations:** Department of Anesthesiology, Tongxiang First People's Hospital, Tongxiang, China

**Keywords:** nephrolithotomy, renal calculi, analgesia, pain, nerve block

## Abstract

**Background:** We aimed to assess the efficacy of intercostal nerve block (ICNB) for pain relief after percutaneous nephrolithotomy (PCNL).

**Methods:** An electronic search of the databases of PubMed, Science Direct, BioMed Central, CENTRAL, Embase, and Google Scholar was conducted. All types of studies conducted on adult patients undergoing PCNL, comparing ICNB with control or any other anesthetic method, and reporting postoperative pain outcomes were included.

**Results:** Six studies were included. Studies compared ICNB with peritubal (PT) infiltration and with control. Pooled analysis of ICNB vs. PT infiltration indicated no difference between the two groups for pain scores at 6–8 h (MD −0.44; 95% CI −3.41, 2.53; I^2^ = 99%; *p* = 0.77), 12 h (MD −0.98; 95% CI −4.90, 2.94; I^2^ = 99%; *p* = 0.62) and 24 h (MD 0.16; 95% CI −0.90, 1.21; I^2^ = 88%; *p* = 0.77). Time for first analgesic demand was also not significantly different between the two groups. Meta-analysis of ICNB vs. control indicated statistical significant difference in pain scores between the two groups at 8 h (MD −1.55; 95% CI −2.60, −0.50; I^2^ = 47%; *p* = 0.04), 12 h (SMD −2.49; 95% CI −4.84, −0.13; I^2^ = 96%; *p* = 0.04) and 24 h (SMD −1.22; 95% CI −2.12, −0.32; I^2^ = 88%; *p* = 0.008). The total analgesic requirement in morphine equivalents was not significantly different between the two groups.

**Conclusions:** ICNB may be effective in reducing postoperative pain after PCNL. However, its efficacy may not be greater than PT infiltration. Current evidence is from a limited number of studies. Further, high-quality randomized controlled trials are needed to provide robust evidence.

## Introduction

Percutaneous nephrolithotomy (PCNL) is a minimally invasive endourological procedure used to manage patients with large, multiple, and staghorn renal calculi (1). The procedure has a higher stone clearance rate as compared to extracorporeal shockwave lithotripsy, with significantly less morbidity as opposed to open surgery (2). However, significant post-operative pain can occur with PCNL in the first 24 h along the nephrostomy tract or due to dilatation of the renal capsule and parenchyma. Recent studies have reported that decreasing the size of the percutaneous tract (miniperc or small-bore PCNL) or completely avoiding the placement of the nephrostomy tube (tubeless PCNL) may help improve post-operative pain scores (3–5).

An alternate method of reducing pain is with the aid of analgesics or regional anesthetic techniques. Non-steroid anti-inflammatory drugs (NSAIDs) and opioids may alleviate post-operative pain in PCNL patients but are associated with several adverse events. Regional anesthesia offers the advantage of direct action at the site of surgery with minimal adverse effects of analgesic drugs. Clinicians have also reported that peritubal (PT) infiltration of the nephrostomy tract with an anesthetic can reduce post-operative pain (6). Amongst nerve blocks, paravertebral, and intercostal nerve block (ICNB) are commonly used to provide post-operative pain relief after PCNL (7, 8).

The role of ICNB as a regional anesthesia technique is well-established in thoracic and abdominal surgery (9, 10). Some authors have also used ICNB for post-nephrectomy pain relief (11). The technique is easy to learn as the nerves travel in neurovascular bundles along the lower border of the ribs. There have been some concerns over complications like pneumothorax with ICNB but the overall incidence is low (12). A number of studies have assessed the efficacy of ICNB for providing pain relief after PCNL (7, 13), but to the best of our knowledge, no review has attempted to systematically analyze level- 1 evidence for its use. Therefore, the purpose of this study was to conduct a systematic literature search and collate data to assess the efficacy of ICNB for pain relief after PCNL.

## Materials and Methods

### Search Strategy

We performed an electronic search of the databases of PubMed, Science Direct, BioMed Central, CENTRAL, Embase, and Google scholar. Databases were searched from inception to 1st July 2020. We used both MeSH terms and free-text keywords for searching relevant articles. Key-words used were, “percutaneous nephrolithotomy,” “intercostal nerve block,” “nerve block,” “analgesia,” and “anesthetic” in various combinations. The search strategy is presented as Supplementary Table S1. The reviewers screened the search results initially by their titles and abstracts for each database. After identifying potentially pertinent articles, full texts of the articles were extracted. Both the reviewers assessed individual articles based on the inclusion and exclusion criteria. Any disagreements were resolved by discussion. After screening, the bibliography of included studies and review articles on the subject were hand searched for any missed references. We conducted this review following the guidelines of the PRISMA statement (Preferred Reporting Items for Systematic Reviews and Meta-analyses) (14).

### Inclusion Criteria

The Population, Intervention, Comparison, Outcome, and Study design (PICOS) framework was used to selection of studies. The review question of interest was: What is the efficacy of ICNB (*Intervention*) vs. control or any other anesthetic method (*Comparison*) for pain relief (*Outcome)* in adult patients undergoing PCNL (*Population)*?

For inclusion in the review, the *population* of the studies was to be adult patients (>18 years) undergoing PCNL. Studies were to study ICNB as the *Intervention* and compare it with control or any other anesthetic method (*Comparison*). *Outcomes* of the study were to include postoperative pain and/or postoperative analgesic consumption. We included all types of studies in this review. Studies were included irrespective of sample size and the type of anesthetic agent used. No restriction was placed on the language of publication. We excluded single-arm studies and studies not reporting relevant data. Furthermore, case series, case reports, and review articles were also excluded.

### Data Extraction

Following mutual agreement on the studies to be included, the two reviewers independently extracted data using a data extraction form. Details of study authors, publication year, study location, study type, sample size, demographic details, operation time, mean stone size/burden, ICNB protocol, control group protocol, and study outcomes were extracted. The primary outcome of interest in our analysis was post-operative pain. The secondary outcomes were total analgesic consumption, time to first analgesic demand, and complications. Outcome data was fed into meta-analysis software and cross-checked for correctness.

### Risk of Bias Assessment

The Cochrane Collaboration risk assessment tool was used for assessing the quality of included studies (15). Two reviewers independently assessed each study. The following seven domains were used for quality assessment: random sequence generation, allocation concealment, blinding of participants and personnel, blinding of outcome assessment, incomplete outcome data, and selective reporting. The study was judged to have “high,” “unclear,” or “low” risk of bias for each domain. Any disagreements were resolved by discussion.

### Statistical Analysis

“Review Manager” (RevMan, version 5.3; Nordic Cochrane Center [Cochrane Collaboration], Copenhagen, Denmark; 2014) was used for the meta-analysis. For this study, similar studies were grouped for the pooled analysis (ICNB vs. PT infiltration and ICNB vs. control). Since all outcomes were continuous variables, they were summarized using the mean difference (MD) with 95% confidence intervals (CI), if measured on the same scale. In case different scales were used, standardized mean difference (SMD) were calculated with 95% CI. We used a random-effects model to calculate the pooled effect size for all our analyses. Heterogeneity was assessed using the I^2^ statistic. I^2^ values of 25–50% represented low, values of 50–75% medium, and more than 75% represented substantial heterogeneity. For studies not reporting continuous variables as median and interquartile range, mean and standard deviation scores were calculated using methods reported by Wan et al. (16). We used the software Engauge Digitizer to extract numerical data if only outcomes were reported only graphically. For total analgesic consumption, data on any other opioids were converted into morphine equivalents for the analysis (17). Due to the inclusion of fewer than 10 studies in the review, funnel plots were not used to assess publication bias.

## Results

**Figure 1:**
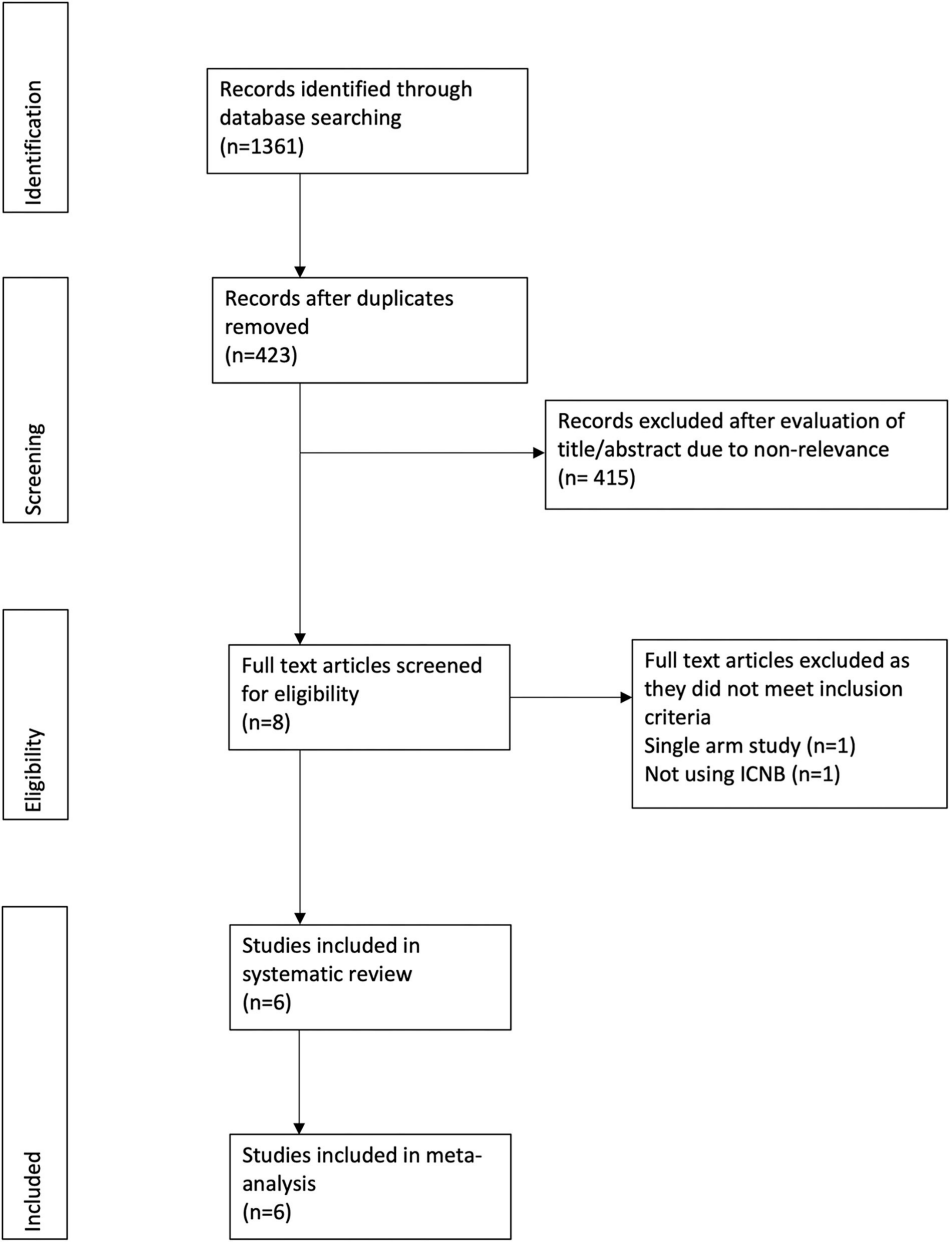
Study flow chart.

The PRISMA flow-chart of the study is presented in Figure 1. A total of 423 unique records were examined. Eight full-texts were reviewed and a total of six studies included in the systematic review and meta-analysis (7, 13, 18–21). Details of the included studies are presented in Table 1. Five were randomized controlled trials (RCTs) (7, 18–21) while one was a prospective non-randomized study (13). The sample size of studies varied from 26–50 patients per arm. No statistically significant differences in baseline variables were reported by any of the included studies. Bupivacaine and ropivacaine were used for the ICNBs. Two studies (7, 18) compared ICNB with PT infiltration, three (13, 20, 21) compared with control while one study (19) was a three-arm trial including ICNB, PT infiltration, and control. All studies administered ICNB after the procedure, except for Ozkan et al. (20).

**Table 1:** Characteristics of included studies.

**References**	**Location**	**Sample size**		**Mean age**		**Male gender (%)**		**Operation time**		**Mean stone size/burden (mm)**		**ICNB group**	**Control group**	**Nephrostomy tube size**
		**ICNB**	**Control**	**ICNB**	**Control**	**ICNB**	**Control**	**ICNB**	**Control**	**ICNB**	**Control**			
Singh et al. (7)	India	32	32	35.8 ± 11.9	36.9 ± 11.3	NR	NR	NR	NR	19.1 ± 5	19.2 ± 6	At 11th, 12th rib with 10 mL of 0.25% bupivacaine at the end of the procedure	PT with 10 cc of 0.25% bupivacaine	24 F
Jonnavithula et al. (18)	India	26	30	41.3 ± 13.4	42.5 ± 11.6	73	70	1.4 ± 0.3 h	1.5 ± 0.2 h	NR	NR	At 10th, 11th, 12th rib with 15 mL of 0.5% ropivacaine at the end of the procedure	PT with 5 mL of 0.5% ropivacaine	14/16 F
Choi et al. (19)	Korea	32	I: 32 II: 32	54 ± 16.2	I: 57.8 ± 13.8 II: 56.5 ± 13	22	I: 22 II: 22	67.6 ± 24.8 min	I: 67.4 ± 22.9 min II: 78.5 ± 41.4 min	302.9 ± 138.6	I: 272.6 ± 129 II: 270.2 ± 144.4	At 10th, 11th, 12th rib with 15 mL of 0.5% ropivacaine with epinephrine at the end of the procedure	I: PT with 20 mL of 0.25% ropivacaine II: No nerve block or infiltration	Tubeless
Ozkan et al. (20)	Turkey	20	20	53 ± 13.6	57.3 ± 7.4	65	70	74.4 ± 23.6 min	76.5 ± 25.4 min	20 ± 6.4	17.5 ± 7.5	At 11th, 12th rib with 8 mL of 0.5% bupivacaine with epinephrine before the procedure	Sham block	Size NR
Honey et al. (21)	Canada	30	33	47.1 ± 8.8	48.7 ± 13.7	60	60.6	NR	NR	30.1 ± 15.7	28.3 ± 12.3	2 ribs above and one rib below the PCNL tract with 20 mL of 0.5% bupivacaine with epinephrine at the end of the procedure	Sham block	8.5 F/6 F, internal. external
Viney et al. (13)	UK	50	50	50 ± NR	48.9 ± NR	52	56	120 min ± NR	115 min ± NR	NR	NR	For three intercostal nerves around the PCNL site with different anesthetic agents at the end of the procedure	Control	Size NR

*ICNB, Intercostal nerve block; NR, not reported; PCNL, percutaneous nephrolithotomy*.

### ICNB vs. PT Infiltration

**Figure 2:**
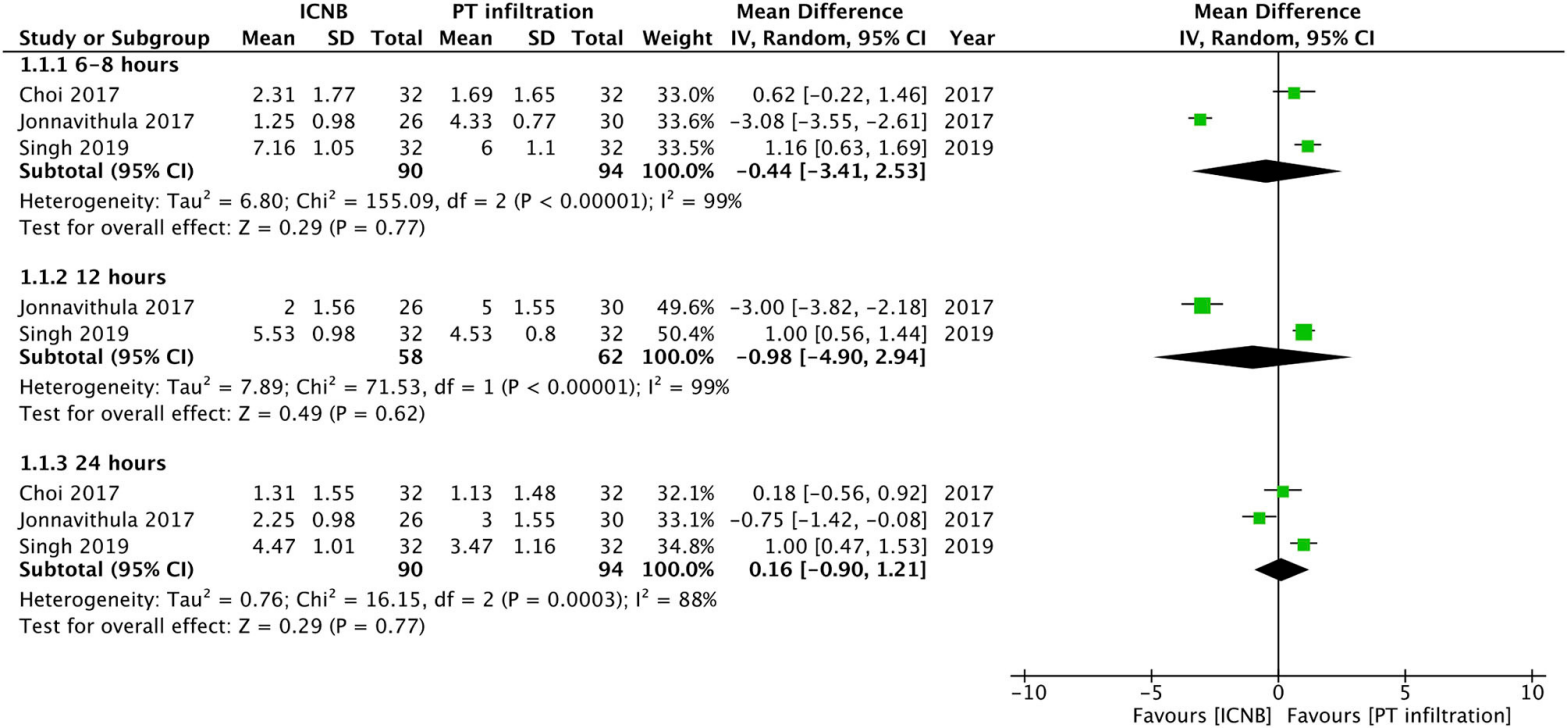
Forest plot of pain scores for ICNB vs. PT infiltration.

**Figure 3:**
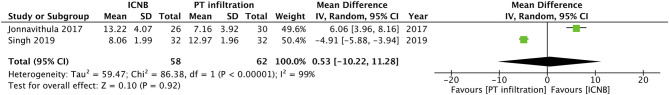
Forest plot of time for first analgesia for ICNB vs. PT infiltration.

Three studies compared post-operative pain on Visual Analog Scale (VAS) between ICNB and PT infiltration. Pooled analysis of pain scores indicated no difference between the two groups at 6–8 h (MD −0.44; 95% CI −3.41, 2.53; I^2^ = 99%; *p* = 0.77), 12 h (MD −0.98; 95% CI −4.90, 2.94; I^2^ = 99%; *p* = 0.62) and 24 h (MD 0.16; 95% CI −0.90, 1.21; I^2^ = 88%; *p* = 0.77) (Figure 2). Time for first analgesic demand in hours was reported by two trials. Meta-analysis indicated no statistical significant differences between the two groups (MD −0.53; 95% CI −10.22, 11.28; I^2^ = 99%; *p* = 0.92) (Figure 3).

Singh et al. (7) analyzed the total diclofenac use between ICNB and PT infiltration groups. The reported a higher total analgesic consumption in patients receiving ICNB as compared to PT infiltration (*p* < 0.001). On the other hand, Choi et al. (19) reported a trend toward lower analgesic consumption (fentanyl) with ICNB as compared to PT infiltration but the result was not statistically significant (*p* = 0.07). None of the studies reported any complications attributable to ICNB or PT infiltration.

### ICNB vs. Control

**Figure 4:**
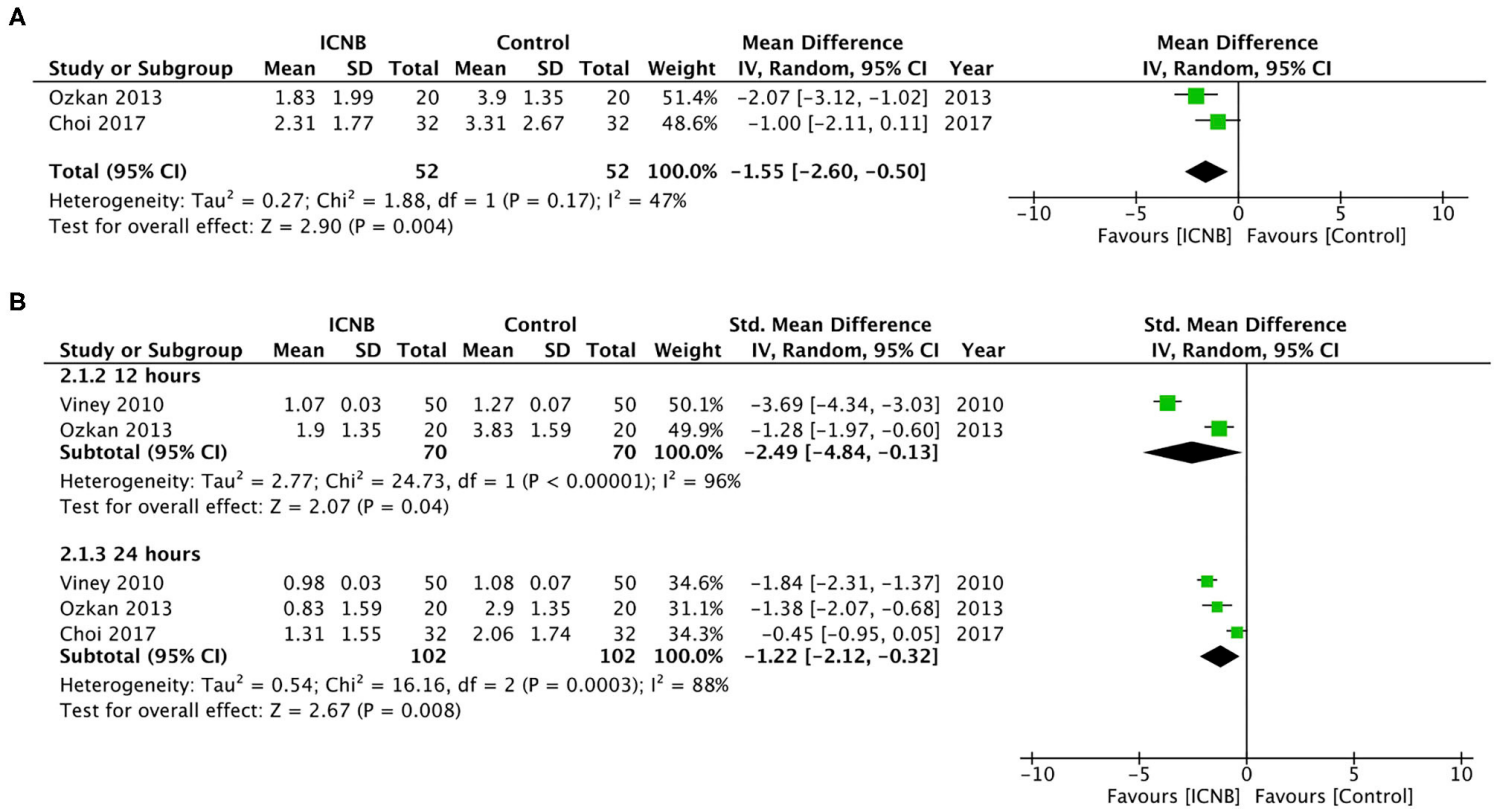
Forest plot of pain scores for ICNB vs. control. **(A)** 8 h **(B)** 12 and 24 h.

**Figure 5:**
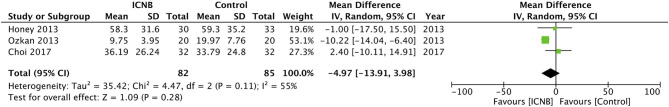
Forest plot of total analgesic requirement in morphine equivalents for ICNB vs. control.

A total of four studies compared outcomes of ICNB with a sham block or no nerve block. Two studies reported postoperative pain outcomes on the VAS scale while one study measured it on a three-point scale. Meta-analysis indicated statistical significant difference in pain scores between the two groups at 8 h (MD −1.55; 95% CI −2.60, −0.50; I^2^ = 47%; *p* = 0.04) (Figure 4A), 12 h (SMD −2.49; 95% CI −4.84, −0.13; I^2^ = 96%; *p* = 0.04) and 24 h (SMD −1.22; 95% CI −2.12, −0.32; I^2^ = 88%; *p* = 0.008) (Figure 4B). Data on the total analgesic requirement in morphine equivalents were pooled from three studies. Two studies reported converted total morphine equivalents themselves, while conversion was required for the study of Ozkan et al. (20). Results indicated no statistical significant difference between the two groups (MD −4.97; 95% CI −13.91, 3.98; I^2^ = 55%; *p* = 0.28) (Figure 5). Viney et al. (13) in their study reported decreased requirement of analgesics in patients receiving ICNB as compared to controls but their results were not statistically significant. Since the study did not report standard deviation scores of the required data, it was not included in this meta-analysis. Time for the first analgesic was not reported by any trial. No study reported complications attributable to ICNB.

### Risk of Bias Analysis

**Figure 6:**
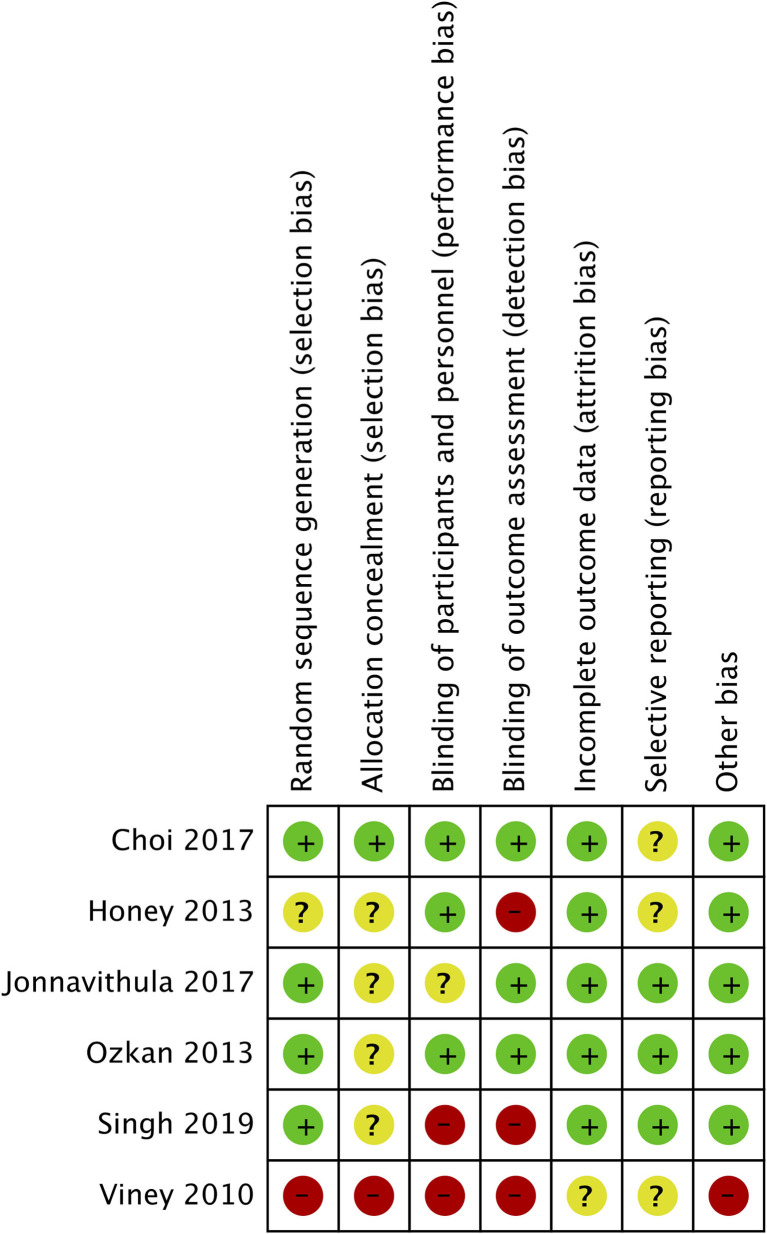
Risk of bias analysis.

The authors' assessment of the risk of bias in the included studies is presented in Figure 6. Adequate methods of randomization were described in four studies (7, 18–20). Appropriate methods of blinding of both participants and outcome assessors were reported by two studies (19, 20). Reporting bias was low with the three RCTs (7, 18, 20) which were pre-registered.

## Discussion

Since its first description, PCNL has practically replaced open surgery for the management of large renal calculi (22, 23). Studies have reported a high stone clearance rate of >90% with PCNL (24, 25). As compared to open surgery, PCNL is associated with reduced operative times, decreased blood loss, shorter hospital stay, and reduced overall complications (23). However, pain after PCNL can be a significant problem in the early postoperative period. No standard published guidelines exists for pain management after PCNL. Clinicians have reportedly used the inter-pleural block, renal capsular block, paravertebral block, and ICNB with varying success (7, 8, 26, 27). While a recent meta-analysis has indicated that paravertebral block is effective in pain management (23), no review has synthesized evidence on the efficacy of ICNB for PCNL to date.

Subcutaneous infiltration of the surgical site with a local anesthetic is a common practice in several surgical specialties, however, its role in PCNL is questionable (28). However, PT infiltration along the entire length of the nephrostomy tract has shown benefits for post-operative analgesia (29, 30). In our analysis comparing ICNB with PT infiltration, we did not find any statistically significant differences between the two techniques for pain scores or time for first analgesic demand. On examination of the forest plots, the study of Jonnavithula et al. (18) was found to favor ICNB while opposite results were reported by Singh et al. (7). Choi et al. (19), on the other hand, found no difference between the two groups. Such variation may be attributed to procedural differences between the three studies. A nephrostomy tube is often inserted after PCNL for unimpeded drainage of the pelvicalyceal system. Studies have reported that smaller tubes are associated with reduced pain (31, 32). In the study of Jonnavithula et al. (18) smaller bore sizes were used as opposed to 24 F tube size in the study of Singh et al. (7). Choi et al. (19) conducted their study on tubeless PCNL. The extent of anesthesia provided by ICNB extends only up to the lateral cutaneous branches of the inter-costal nerves which innervate the access site of PCNL (33). The nerve supply of the deeper abdominal viscera is dependent on the celiac plexus (34). Thus, it may be postulated that in the study of Singh et al. (7) using larger nephrostomy tubes, PT infiltration may have provided better analgesia due to direct action of the anesthetic along the entire nephrostomy tract. This effect may have not been significant in the remaining two studies with tubeless (19) or small-bore PCNL (18).

In the second part of our study, we compared outcomes of ICNB with control and found significantly reduced pain scores in patients receiving ICNB at 8, 12, and 24 h. However, the total analgesic requirement was not significantly different between the two groups. It is important to note that the effect size for pain scores at all three time intervals was small with the upper end of 95% CI very close to 0. Similar small effect sizes have been reported by Tan et al. (8) in their meta-analysis of paravertebral blocks for PCNL (Analgesic requirement: SMD −1.55; 95% CI −2.18, −0.92). In comparison with other surgeries, Detterbeck et al. (35) in a systematic review have reported significantly better outcomes with ICNB as compared to oral analgesics for thoracotomy patients. Their study indicated that continuous ICNB with a catheter can provide better results as compared to single ICNB. While catheter placement may be feasible in thoracotomy where surgical access is available, it may significantly increase morbidity after PCNL (13). Furthermore, being a minimally invasive procedure, pain intensity, and duration after PCNL is comparatively less.

As with any regional anesthesia procedure, ICNB may also result in complications. Experimental studies on healthy volunteers have indicated reduced vital capacity with ICNB (36), but these results have not been corroborated by other studies (37). Other complications with ICNB may include pneumothorax, pleural effusions, abscess formation, neuritis, and hypotension. The reported incidence of pneumothorax with ICNB has been quite variable ranging from 0.073 to 19% (38). In our review, none of the included studies reported any complication attributable to ICNB. This may be due to the limited sample size of all included trials.

Our review has some limitations. Foremost, a limited number of studies with small sample size were available for data analysis in our review. Secondly, there were inter-study methodological differences that may have contributed to the high heterogeneity in our analysis. There were differences in the anesthetic agents, the use of epinephrine, utilization and size of nephrostomy tubes, mean stone size/burden, number of nerves blocked, timing of ICNB etc. These may have skewed results of our analysis. Due to lack of comparative studies between ropivacaine and bupivacaine for ICNB, our study could not comment on the superiority of one agent over the other. However, both ropivacaine and bupivacaine are long-acting anesthetic agents with similar pharmacokinetic properties (39). Comparative studies for other regional anesthesia techniques have indicated no difference between the two agents (39, 40). Lastly, the outcomes of any regional anesthesia technique are also dependent on the skill of the operator and the pain threshold of the patient. These factors could not be accounted for in our review.

To conclude, the results of our study indicate that ICNB may be effective in reducing postoperative pain scores in patients undergoing PCNL. However, its efficacy may not be >PT infiltration. Current evidence is from a limited number of studies. There is a need for further trials with large sample size comparing ICNB with control and PT infiltration to establish high-quality evidence.

## Data Availability Statement

The original contributions generated for this study are included in the article/Supplementary Material, further inquiries can be directed to the corresponding author/s.

## Author Contributions

TC conceived, designed the study, and involved in the writing of the manuscript. ZZ and JD collected the data, and performed the literature search. All authors have read and approved the final manuscript.

## Conflict of Interest

The authors declare that the research was conducted in the absence of any commercial or financial relationships that could be construed as a potential conflict of interest.
