# Supplementary material for: Efficacy of Intercostal Nerve Block for Pain Control After Percutaneous Nephrolithotomy: A Systematic Review and Meta-Analysis
Source: Front Surg. 2021 Jan 28;8:623605. doi: 10.3389/fsurg.2021.623605 (PMC7876386; doi:10.3389/fsurg.2021.623605)
Supplement: Supplementary file 1 [file Table_1.DOCX]

Supp Table S1. Search strategy and results of PubMed database

| **Search number** | **Query** | **Search Details** | **Results** |
| --- | --- | --- | --- |
| **1** | (percutaneous nephrolithotomy) AND (intercostal nerve block) | ((("nephrolithotomy, percutaneous"[MeSH Terms] OR ("nephrolithotomy"[All Fields] AND "percutaneous"[All Fields])) OR "percutaneous nephrolithotomy"[All Fields]) OR ("percutaneous"[All Fields] AND "nephrolithotomy"[All Fields])) AND ((((("intercostal nerves"[MeSH Terms] OR ("intercostal"[All Fields] AND "nerves"[All Fields])) OR "intercostal nerves"[All Fields]) OR ("intercostal"[All Fields] AND "nerve"[All Fields])) OR "intercostal nerve"[All Fields]) AND (((("block"[All Fields] OR "blocked"[All Fields]) OR "blocking"[All Fields]) OR "blockings"[All Fields]) OR "blocks"[All Fields])) | 8 |
| **2** | (percutaneous nephrolithotomy) AND (nerve block) | ((("nephrolithotomy, percutaneous"[MeSH Terms] OR ("nephrolithotomy"[All Fields] AND "percutaneous"[All Fields])) OR "percutaneous nephrolithotomy"[All Fields]) OR ("percutaneous"[All Fields] AND "nephrolithotomy"[All Fields])) AND (("nerve block"[MeSH Terms] OR ("nerve"[All Fields] AND "block"[All Fields])) OR "nerve block"[All Fields]) | 22 |
| **3** | (percutaneous nephrolithotomy) AND (analgesia) | ((("nephrolithotomy, percutaneous"[MeSH Terms] OR ("nephrolithotomy"[All Fields] AND "percutaneous"[All Fields])) OR "percutaneous nephrolithotomy"[All Fields]) OR ("percutaneous"[All Fields] AND "nephrolithotomy"[All Fields])) AND (("analgesia"[MeSH Terms] OR "analgesia"[All Fields]) OR "analgesias"[All Fields]) | 121 |
| **4** | (percutaneous nephrolithotomy) AND (anaesthetic) | ((("nephrolithotomy, percutaneous"[MeSH Terms] OR ("nephrolithotomy"[All Fields] AND "percutaneous"[All Fields])) OR "percutaneous nephrolithotomy"[All Fields]) OR ("percutaneous"[All Fields] AND "nephrolithotomy"[All Fields])) AND (((((((((((((((((((((("anaesthetically"[All Fields] OR "anaesthetics"[All Fields]) OR "anesthetics"[Pharmacological Action]) OR "anesthetics"[MeSH Terms]) OR "anesthetics"[All Fields]) OR "anesthesiology"[MeSH Terms]) OR "anesthesiology"[All Fields]) OR "anaesthetise"[All Fields]) OR "anaesthetised"[All Fields]) OR "anaesthetising"[All Fields]) OR "anaesthetization"[All Fields]) OR "anaesthetize"[All Fields]) OR "anaesthetized"[All Fields]) OR "anaesthetizing"[All Fields]) OR "anesthetic s"[All Fields]) OR "anesthetically"[All Fields]) OR "anaesthetic"[All Fields]) OR "anesthetic"[All Fields]) OR "anesthetization"[All Fields]) OR "anesthetize"[All Fields]) OR "anesthetized"[All Fields]) OR "anesthetizes"[All Fields]) OR "anesthetizing"[All Fields]) | 178 |
